# Prehospital administration of surfactant to a premature neonate in respiratory distress

**DOI:** 10.1186/s13049-019-0664-9

**Published:** 2019-10-29

**Authors:** Charles Haviland Mize, Lhab Dorji, Ken Zafren

**Affiliations:** 1 Bhutan Emergency Aeromedical Retrieval, Emergency Department, Jigme Dorji Wangchuck National Referral Hospital, Thimphu, Bhutan; 2 Department of Emergency Medicine, Alaska Native Medical Center, Anchorage, AK USA; 3 Department of Emergency Medicine, Stanford University Medical Center, Stanford, CA USA; 4 International Commission for Mountain Emergency Medicine (ICAR MEDCOM), Zürich, Switzerland

**Keywords:** Surfactant, Altitude, Neonatal respiratory distress, Prehospital care, HEMS

## Abstract

The population of the Kingdom of Bhutan is scattered in small villages throughout the eastern Himalaya. Infants born prematurely in villages have no access to neonatal intensive care until they are transported to the national referral hospital, a process that once took hours, if not days. After the introduction of a helicopter critical-care retrieval team, we were able to send a trained team to a remote location that successfully administered surfactant and initiated critical care to a premature, extreme low birth weight infant in severe respiratory distress in the first hour of life. Although the infant was in shock and in a near-arrest state at the time the team arrived, he made an excellent recovery after resuscitation by the team.

## Introduction

Premature neonates are at high risk of suffering respiratory distress syndrome (RDS) soon after birth. The incidence of RDS correlates strongly with severity of prematurity. Approximately 91% of neonates born at 23–25 weeks gestation will experience RDS compared with 52% of those born at 30–31 weeks gestation [1, 2]. Surfactant deficiency is the primary cause of RDS. Produced by type-2 alveolar cells (pneumocytes), surfactant is a lipoprotein that acts to reduce alveolar wall tension and allow lung expansion without atelectasis. Without surfactant, each exhalation causes collapse of small airspaces, with subsequent exhalations leading to progressively severe atelectasis [2]. Type-2 alveolar cells differentiate and begin synthesis of surfactant between 24 and 28 weeks gestation. Administration of steroids prior to birth is an important and proven means of preventing RDS [3].

The cornerstone of treatment for RDS is the administration of exogenous surfactant. Since its introduction in the late 1980s, exogenous surfactant has been proven to save infant lives in more than 30 randomized clinical trials involving more than 6000 infants. Early surfactant administration has been demonstrated to reduce mortality, severity of lung disease, need for mechanical ventilation and the risk of chronic lung disease [4–6]. When infants are born far from tertiary care hospitals, however, early administration of surfactant can be impossible [7]. Early administration of surfactant can enable an infant to survive until transfer to a NICU [8]. In the Kingdom of Bhutan, the population is scattered in small villages throughout the eastern Himalaya. Although the Kingdom of Bhutan has established ‘basic health units’ - BHUs - in most villages and towns throughout the country, these BHUs are not equipped to provide emergency medicine or critical care. BHUs are staffed with a single ‘health attendant,’ someone trained to assist with normal childbirth, vaccinations, preventative care and to assist in referrals, or by either a nurse or a physician with a single year of post-graduate training. Each BHU can send patients to a district hospital manned by a few physicians and usually, but not always, by one or two specialists (in either internal medicine or ob/gyn, but rarely both). Each of Bhutan’s 20 districts has a District Hospital, sometimes two: there are 29 district hospitals. These hospitals do not have critical care or surgical capacity and do not provide modern emergency medicine or critical care. These district hospitals refer in turn to one of three regional referral hospitals - the only hospitals in the country with intensive care medicine, emergency medicine and surgery. Only one of these referral hospitals - the Jigme Dorji Wangchuck National Referral Hospital, in the capital Thimphu - has specialists and subspecialists in nearly all fields, has an emergency department with emergency specialist physicians, an intensive care unit and a neonatal intensive care unit. There are only 345 physicians in the country for a population of approximately 900 thousand and most of these physicians work in the regional referral hospitals. Although Bhutan is training its first class of paramedics, at present ambulances are generally staffed with a driver alone, and the lack of road access coupled with small roads that are often impassable during monsoon render transfer between BHUs, District Hospitals and referral hospitals unreliable [9]. Infants born prematurely in villages have no access to neonatal intensive care until they are transported to the national referral hospital, a process that once took hours, if not days. Determined to care for all of its citizens and to provide critical care medicine throughout the country, Bhutan created a helicopter-deployed critical care and resuscitation team.

## Case report

In March 2018, a boy was born in a small town (population 6000) in the mountains of Bhutan at an elevation of 2500 m. He was born at 28 weeks gestational age by uncomplicated vaginal delivery to a healthy, HIV-negative mother who had received antenatal care at the local health clinic. The infant weighed 0.95 kg at birth and was initially vigorous (Apgar score 9/10) but quickly developed respiratory distress. A local physician evaluated the infant and then called for helicopter evacuation by the critical care retrieval team. The team, consisting of a physician specialist in emergency medicine and an emergency nurse, arrived within 1 h of the infant’s birth. At the time the team arrived, he was tachypneic (rate 60+ breaths/min), bradycardic (75–80 beats/min), normothermic (37 °C rectal) and had a blood glucose of 90 mg/dl. Oxygen saturation (SpO_2_) recorded on right and left arm hovered around 70% while on 2 L oxygen by nasal cannula.

Given the severity of the infant’s condition, time since birth and the fact that changes in weather would extend the time required to transfer the infant by helicopter to the neonatal intensive care unit (NICU) in Bhutan’s capital (2 h as opposed to 30 min), the team decided to undertake prehospital resuscitation and to consider administration of surfactant. While preparing intubation equipment and surfactant, the infant was first given positive pressure ventilation (PPV) with a bag-valve mask for several minutes, which brought an improvement in SpO_2_ to 80%. When the oxygen saturation failed to rise despite several minutes of PPV, the team then intubated the infant with a Macgrath video MAC laryngoscope. Correct placement of the endotracheal (ET) tube was confirmed with capnography. The infant was then placed in the left lateral decubitus position and 1 mL (80 mg) of surfactant (poractant alfa) was administered via syringe and iv cannula through the ET tube. He was then returned to the supine position, ventilated for 1 min (60 breaths), then placed in the right lateral decubitus position and another 1 mL (80 mg) of surfactant was administered. The infant was returned to the supine position and ventilation continued. Within the next 2 min, his heart rate rose to 120 beats/min and SpO_2_ rose to 97%. The team took care to observe changes in the infant’s lung compliance to prevent iatrogenic injury during mechanical ventilation. The team then positioned the mother on board the helicopter to allow for “kangaroo-mother-care” (skin-to-skin contact) for transport to the NICU. The infant’s endotracheal tube became dislodged accidentally by head movement during positioning on his mother’s chest. The patient had received approximately 15 min of PPV via endotracheal tube at the time of extubation. The team elected to defer re-intubation because the heart rate, respiratory rate and pulse oximetry remained normal, and because the procedure of ‘intubation – surfactant administration – extubation’ (InSurE procedure) is a recognized and established means of surfactant administration [5]. The team was prepared to re-intubate during flight if there were signs of deterioration.

The infant had an uneventful flight to the NICU (altitude 2000 m). Approximately 10 h after NICU admission, he developed recurrent hypoxia to 85%, which responded to repeat dosing of surfactant followed by a few hours of mechanical ventilation and then continuous nasal positive airway pressure (CPAP) for 4 days. He was discharged from the NICU to the Kangaroo Mother Care Unit (the nursery) in good health 1 month after admission. He was discharged from the hospital, still in good health about 3 weeks later.

## Discussion

Early administration of surfactant to premature, low-birth weight infants in respiratory distress is a well-established, critical treatment for neonatal RDS. In the developing world, however, the availability of this treatment is often limited by poor access to surfactant and a lack of expertise and equipment needed to administer it. To our knowledge, this is the first recorded case of successful prehospital surfactant administration by a critical care retrieval team.

## Conclusion

Prehospital administration of surfactant can be performed successfully by a prehospital critical care retrieval team, even at moderately high altitude in a developing country.

## Data Availability

Data sharing not applicable to this article as no datasets were generated or analyzed during the current study.

## Abbreviations

CPAP: Continuous Positive Pressure Ventilation
InSurE Procedure: Intubation – surfactant administration – extubation
NICU: Neonatal intensive care unit
PPV: Positive pressure ventilation
RDS: Respiratory distress syndrome
